# Addiction of High Dose of Benzodiazepine: Verona Detox Approach With Flumazenil

**DOI:** 10.3389/fpsyt.2022.857376

**Published:** 2022-03-31

**Authors:** Rebecca Casari, Antonio Metastasio, Lorenzo Zamboni, Martino Biasioli, Simone Campagnari, Fabio Lugoboni

**Affiliations:** ^1^Unit of Addiction Medicine, Department of Internal Medicine, Integrated University Hospital of Verona, Verona, Italy; ^2^Camden and Islington NHS Foundation Trust, London, United Kingdom; ^3^Department of Neurosciences, University of Verona, Verona, Italy

**Keywords:** benzodiazepine, addiction, flumazenil, detox, high dose

## Abstract

**Introduction:**

Since the 1990s there has been evidence of the significant role Flumazenil (FLU) has in benzodiazepines (BZD) detoxes. The Verona Detox approach has been developed for high dose BZD and Z-drug detoxification via continuous subcutaneous infusion of FLU, a selective BZD receptor antagonist acting on the BZD subunit of the GABA-A receptor. Flumazenil is licensed in the United Kingdom and other countries to treat only BZD overdose although numerous studies have demonstrated its effectiveness in rapidly resetting GABA-A receptors, quickly reducing tolerance and dependence from BZD, and providing a safe and rapid detox from benzodiazepines.

**Objective:**

The aim of this article is to provide all healthcare professional who are interested in BZD detoxification with an approach and clear practical information on how to administer FLU.

**Method:**

In this article we outline the approach in detail, describing all medical and nursing procedures day by day. This detox treatment is indicated for patients abusing from at least 5 Defined Daily Dose (DDD) of BZDs or Z-drugs. The process lasts 7 days, and is conducted under medical supervision (daily reviews) and continuous nursing (24/7). During this period, 7mg of FLU is administered (1 mg/24) through an elastomeric pump, via continuous subcutaneous infusion.

**Conclusion:**

To this day, the largest database of FLU detoxification was published by our group, showing how this treatment is safe, with very little side effects even in patients with significant medical comorbidities.

## Introduction

The use of benzodiazepines (BZD) has an history of more than 60 years. The first BZD, chlordiazepoxide, was approved in 1960, primarily to treat anxiety and insomnia.

The 1960s saw a rapid increase of the use of this drug, soon joined by the still popular diazepam and, gradually, by several others.

In the 1970s, diazepam became the best-selling drug in the United Kingdom (United Kingdom) and the United States of America (United States). Since BZDs can give tolerance and dependence even in a short period, their use was recommended for a very limited time (2-4 weeks) (1). These recommendations were largely disregarded by doctors and patients themselves. Indeed, it is difficult to ignore the rapid benefits of these drugs coupled with such limited side effects. One of the keys to understanding the widespread use of BZD is in fact its substantial lack of acute toxicity. However, the chronic use causes a number of serious side effects: cognitive impairment, falls, traffic accidents and dependence (1).

Benzodiazepines tolerance has been reported already in 1961 (2), but this and other reports during the 1960s and 70s have been “covered” by the enthusiastic use of these drugs, which were able to replace barbiturates.

Benzodiazepines tolerance has some specific characteristics compared with other drugs of abuse. The very low toxicity of BZDs and their high potential of tolerance can induce the consumption of extremely high doses (3–5).

One of the major obstacles which has prevented an appropriate analysis of BZD dependence is that many (both doctors and patients) considered that, in a world of increasing anxiety and sleeplessness, spending years taking 1-2 pills per day is more acceptable than fighting with anxiety and insomnia. As a consequence, long-term use of BZD is a phenomenon that affects between 2 and 7.5% of the population in developed countries (1, 6, 7).

Some of these users take high doses of BZD (HDUs). These patients are summarily categorized as patients with major psychiatric disorders or drug addict. This is inaccurate, because a significant portion of them does not have any major psychiatric disorders.

Few epidemiological studies have analyzed the problem of HDUs. In a population based cross-sectional study with 520,000 swiss patients, it was estimated that 1.6% used BZDs in very high doses, exceeding the maximal recommended daily dose more than twice (8). Notably, surveys from France, Germany, Italy, and United Kingdom carried out during the 1990s showed that 3.9% and 3.2% of current hypnotic and anxiolytic users had been taking a higher dose than the recommended therapeutic range (1).

The severe discomfort experienced by patients trying to stop BZD use led to the development of treatment strategies for discontinuing these medications. The common management of BZD withdrawal syndrome includes, either individually or in combination: (i) a gradual tapering of the drug; and (ii) switching to an equivalent dose of a long half-life BZD before tapering withdrawal (7). If properly applied, the tapering approach works in long-term users, but it has very little success in HDUs (1, 9, 10).

BZD withdrawal from high dose usage is clinically relevant, very poorly tolerated and risky for the patient’s health. In some cases, it can lead to major events such as (potentially lethal) seizures (1, 11). For HDUs with personality disorder and/or co-dependency on alcohol and illicit drugs, the idea of using substitution treatment is currently taking hold (by prescribing slow onset of action BZDs such as clonazepam), which is an approach similar to how this is used with methadone for heroin users (12).

For HDUs without co-dependency and with less severe psychiatric comorbidities, the traditional approach of BZD tapering is characterized by high dropout and relapse rates. In these cases, the detoxification by Flumazenil (FLU) infusion should be the primary approach (3, 9, 13).

## Scientific Evidence of Benzodiazepine Detox Using Flumazenil

Since the 1990s there has been evidence that FLU is safe and can play a significant role in BZD detoxes. For instance, a study on patients dependent on lormetazepam showed that FLU administration as i.v. infusion did not provoke severe withdrawal but caused a normalization of the BZD receptor sensitivity (14). A few years later, the same research group undertook the first randomized, placebo-controlled study comparing FLU i.v. (1 mg twice a day) infusion versus the usual tapering off detox with oxazepam. FLU immediately reversed BZD effects on balance task and significantly reduced withdrawal symptoms in comparison with oxazepam and placebo on both self-reported and observer-rated withdrawal scales. The partial agonist also reduced craving scores during the detox procedure. In addition, during oxazepam tapering, patients experienced paradoxical symptoms that were not apparent in FLU patients. Patients treated with FLU showed significantly lower relapse rates on days 15, 23, and 30 after detox week (3). A similar approach, based on subcutaneous administration of FLU, with similar results, was undertaken in Australia (4, 15). Furthermore, the Australian team used this approach to treat a patient with hypersomnia (16). The largest database, to the best of our knowledge, on FLU detoxification was published by our group (5, 17) and showed that this treatment is safe with very little side effects even in individuals with significant medical comorbidity.

## Benzodiazepine Detox With FLU

### Scope

This approach has been developed for high dose BZD and Z-drug detoxification via continuous subcutaneous infusion of FLU. FLU is a selective BZD receptor antagonist acting on the BZD subunit of the GABA-A receptor. FLU is licensed in the United Kingdom and other countries to treat BZD overdose although numerous studies have demonstrated effectiveness in rapidly resetting GABA-A receptors (it is suggested at a rate of 5% per day), quickly reducing tolerance and dependence from BZD, and providing a safe and rapid detox from benzodiazepines. The severity of BZD misuse is measured by the Defined Daily Dose (DDD), which according to the World Health Organization Collaborating Center for Drug Statistic Methodology is “the assumed average maintenance daily dose of BZD when used for its main indication in adults” (18). For Diazepam it is 10 mg. This detox treatment is indicated for patients with at least 5 DDD BZDs (5, 19) and Z drugs (20, 21), although the treatment is more effective for individuals assuming significantly higher doses (10 DDD or more). The equivalence between BZDs and Z drugs is given in Supplementary Appendix 1.

### Inclusion and Exclusion Criteria

Patients suitable for this procedure include:

• Patient dependent on BZDs and/or Z-drugs for at least six months at an equivalent dose of diazepam (EDD) of more than 50 mg/day, with or without psychiatric comorbidities and other addictions (including alcohol) (5), who alone or with their physician’s help, have tried unsuccessfully to discontinue BZDs due to a severe withdrawal syndrome;
• Delayed BZD withdrawal syndrome may also benefit from this treatment;
• Age ≥ 18 years;
• Epilepsy, psychiatric comorbidities, cross addictions (e.g., alcohol + BZDs) and cognitive dysfunction affecting capacity are not contraindications but need particular attention during the initial assessment (5, 22) or use of Mental Capacity Act 2005 parameters where indicated. Clonazepam and antiepileptic therapy prior or during the procedure (see below) reduce the risk of seizure or epilepsy (5, 11, 22);
• In case of opioid abuse, adequate replacement therapy must be in place (5);
• In the case of comorbid alcohol and BZD dependence/misuse it is advisable to proceed with alcohol detoxification first (with BDZ), and consider longer FLU treatment (14–21 days);
• In cases of cocaine abuse there are no pharmacological treatment guidelines. We treat cocaine abusers using N-acetylcysteine (23), an aftercare treatment (rehab) is mandatory after the recovery;
• In cases of cannabinoids abuse there are no guidelines. After recovery, an aftercare treatment is mandatory with a medical and psychological intervention;
• In major depression or personality disorder, a psychiatric consultation must be performed in order to be sure that the patient is stable and capable of undergoing detox;
• Medical comorbidities – for example, severe obesity, ischemic heart disease (IHD), diabetes, liver impairment – are not a contraindication but need specific monitoring during treatment (and need to be planned during the initial assessment) (5);
• Patients with less severe BZD dependence (daily intake of less than five times the DDD) are directed to their general practitioner in the first instance.

Exclusion criteria include:

• Pregnancy or breast feeding;
• Lack of written consent;
• Substance Use Disorder (illicit drugs) with no current treatment or plan to treat in the near future;
• Subjects diagnosed with psychosis without any treatment in place.

### Approach Equipment

• FLU – order by brand name and posology (Anexate 1fl 0,5 mg/5 ml);
• Elastomeric pump – es Baxter Infusor LV1,5 (cod. 2C1087KP);
• Saline solution;
• Other equipment;
• BP machine;
• O_2_ pulsimeter;
• Thermometer;
• Blue butterfly needles;
• A sling for elastomeric pump;
• Alcohol wipes;
• Adhesive dressing to attach needle to skin;
• 10 ml or 20 ml syringe;
• Green needle;
• IV cannulae Medication;
• Levetiracetam 250 mgs and 500 mg tabs (if patient has not taken sodium valproate prophylactively **2/52 pr**ior to detox);
• Clonazepam 0,5 mg and 2 mg tabs;
• Diazemuls 10 mls.

## Pre-Detoxification Assessment and Management

(a) Start sodium valproate CHR 500 mgs once a day for 7 days, then 500 mgs BD as prophylactic treatment against seizures (the medication should be started at least 14 days before day 1 of detox). Patients who do not tolerate sodium valproate or it is contraindicated will be prescribed levetiracetam 250 mgs BD on the day of the detoxification.

Sodium Valproate (2-n-propylpentanoic acid) is an antiepileptic drug traditionally used for the treatment of seizures. Its mechanism of action involves an increase in γ-aminobutyric acid (GABA)-ergic and glutamatergic neurotransmission (24).

It remains a popular drug due to its proven therapeutic benefits with a low cost; it shows efficacy, with a good safety profile and a relatively low drug–drug interaction (25).

Evidence suggests that levetiracetam is as effective as Sodium Valproate for the cessation of Status epilepticus in adults. It has favorables effect profile, logistics of administration, drug cost, inclusion on hospital formularies, and drug availability (26). Based on these evidences, our group decided thate these two would be the antiepileptic drugs of choice.

(b) Full Psychiatric assessment (including, where indicated, psychometric testing: Mini Mental State Evaluation (MMSE) (or Addenbrooke’s Cognitive Examination – ACE III if necessary), Hamilton depression rating scale, Hamilton Anxiety scale, Brief Substance Craving Scale, Alcohol Use Disorder Identification Test (AUDIT).
(c) Physical examination.
(d) electrocardiogram (ECG).
(e) Blood test (FBC, U&Es, LFTs).
(f) Other investigation if requested by the assessing physician.

### Day 1

(a) Medical review of patient and informed consent signed.
(b) The detox process is conducted under medical supervision (daily reviews) and continuous nursing (24/7).
(c) BZDs withdrawal scale (CIWA-B) (see Supplementary Appendix 2) and/or Brief Substance Craving Scale (see Supplementary Appendix 3) is administered daily.
(d) If Sodium Valproate has not been taken prophylactively prescribe levetiracetam 250 mgs twice a day (BD). Prescribe 5 mgs (1 mg during the day + 4 mgs nocte) clonazepam *per os* (PO).
(f) Pro re nata (PRN) Mirtazapine 15 mgs nocte for insomnia (Patients with comorbid anxiety respond better to the period of withdrawal than patients where insomnia is the main co-morbid symptom).
(g) Nursing staff fill the Baxter 2C1087KP elastomeric pump with 14 vials of 0.5 mg/5 ml of FLU diluted in 190 ml of saline solution as per training received and per film. Place pump in a collar and the sling around neck.
(h) Consider an IV cannula for emergency treatment.
(i) Attach a blue butterfly needle to tube from the elastomeric pump. The needle and the last part of the tube from the pump should be attached to the patient’s skin in order to remain at a constant temperature of 31-32 C degree. The pump should be placed at the same height of the needle with within ± 10-15 cm.
(j) Start continuous subcutaneous infusion of FLU. 1 mg/24 h will take 1 week at an infusion rate of 1.5 ml/hr, which is automatically delivered by the pump.
(k) The patient is shown and instructed to check the infusion site regularly making sure that the area is dry and clean, and that the needle is connected to the elastomeric pump and the solution is flowing.
(l) The nursing staff will review the patient every 6 hours (or more often if necessary) checking blood pressure, pulse rate, temperature and O_2_ saturation and will also make sure that the area of injection is clean and dry.
(m) Psychological support (cognitive behavioral approach) is recommended to all patients during the recovery.

### Managing Seizures

Seizures have been found to be rare (incidence ≤ 0.2%); both cases in the study had significant medical problems, namely epilepsy and in 1 of the cases the seizure was not clearly established. In the unlikely event of a seizure, administer diazepam 10 mgs IV or per rectum (PR) up to x3 and stop subcutaneous infusion. The patient is managed as per standard protocols for seizure management (see Supplementary Appendix 4) and eventually transferred to closest designated emergency department for physical examination, investigations and possible admission for observation. Whether to continue with the detox at a slower rate will depend on numerous factors e.g., nature of seizure, pre-existing medical conditions.

#### Day 2-6

(a) Daily review by supervising physician.
(b) Continue sodium valproate (500 mg BD) or increase levetiracetam (day 2: 750 mg; day 3: 500 mg BD; then continue 500 mg BD).
(c) Gradual tapering off of clonazepam at the rate of 0.5-1 mgs per day (according to patient’s needs).
(d) Consideration of sedative antihistaminic if sleeping problems emerge or persist.
(e) Administration and review of the Clinical Institute Withdrawal Assessment Scale (CIWA-B) BZDs withdrawal scale.
(f) Check subcutaneous site is dry, clean and that infusion flow is uninterrupted.
(g) The nursing staff will review the patient every 6 hours (or more often if necessary) checking blood pressure, pulse rate, temperature and O_2_ saturation, and will also make sure that the area of injection is clean and dry.

#### Day 7 (or 7-14 If Extended Detox Indicated)

(a) Review by supervising physician.
(b) The nursing staff reviews patient every 6 hours (or more often if necessary) checking blood pressure, pulse rate, temperature and O_2_ saturation and will also make sure that the area of injection is clean and dry.
(c) Administration and review of the CIWA-B BZDs withdrawal scale.
(d) If symptoms persist or BZD taper has not finished, extend detox for another week repeating days 1-7 above.
(e) Gradual tapering off of clonazepam at the rate of 0.5-1 mgs per day or slower if necessary (according to patient’s needs) in the second week.
(f) If BZD withdrawal symptoms have disappeared and patient has also ended the BZD taper, detox is terminated.
(g) Patient is discharged with no clonazepam.
(h) If patient wants to discharge after 1 week and is on clonazepam 1 mg or less, advice to taper off at a rate of 0.25 mgs every two weeks or slower (if necessary).

**After FLU, continue antiepileptics drug, riducing the dosage slowly and stop in 30-40 days.**

#### Day 7-21

• In case of patients with more severe addictions that have relapsed a previous FLU detoxification (20), for other clinical reasons (severity of the BZD addiction and/or psychiatric comorbidity) or for patient’s personal choice, the infusion can be prolonged to 14 or 21 days.
• Repeat steps Days 1-7 above.
• Our case reviews seem to also suggest that longer detoxes have better long-term outcomes (5, 19). In this case the procedure is the same of day 2 to 6.

### Follow Up

Outpatient psychiatric review after 15 and 30 days (e.g., recommendations for treatment of ongoing medical/psychosocial factors).

Patient discharged with residual clonazepam treatment will undergo a gradual tapering at the rate of 0.25 mgs every 14 days (most of the patient will not need to be discharged with clonazepam).

A follow up call with psychiatrist after 3 months of treatment is arranged.

## Limitations

There are some limitations:

• The treatment is only for mono-abusers patients;
• The treatment is feasible only with an hospital recovery.

## Discussion

Benzodiazepines are among the most commonly prescribed psychotropic medications worldwide. The possibility that chronic use of BZD can cause deficits in learning, attention, memory, depression and occurrence of injurious falls, road traffic and other accidents, has been well known for a long time. The risk of dependence after long-term use has been largely described, as reflected in the appearance of a series of symptoms when the drug is abruptly withdrawn.

In addition to subjects that begin using BZD to treat anxiety and insomnia disorders and end up using them inappropriately, there are subjects that deliberately abuse BZD. In this case, BZD are taken in an attempt to fight anxiety or increase the effect of other drugs such as alcohol or opioids, this being part of a poly-drug use pattern (5, 27).

The withdrawal syndrome, even from regular therapeutic doses of BZD, can be severe and, in some cases, may preclude the patient from ceasing the use of the drug (1, 28). The severe discomfort experienced by patients stopping long-term BZD use initiated the development of treatment strategies for discontinuing these medications. The common management of BZD withdrawal syndrome include, either individually or in combination: (i) a gradual tapering of the drug; (ii) switching to an equivalent dose of a long half-life BDZ before tapering withdrawal; (iii) adding medications prior to detoxification and continuing those medications after discontinuation, and (iv) psychotherapy (cognitive behavioral therapy and other approaches) (28). In a fairly recent review, the levels of evidence of efficacy of all these approaches were assessed. They were high for gradual withdrawal over a period of several weeks or months, moderate for adding some concomitant pharmacotherapy for BZD withdrawal (carbamazepine, antidepressants, antihistaminergic drugs, melatonin), but low for other drugs such as pregabalin and beta-blockers; also low for switch to a long-acting BZD (diazepam) (7). In the same review, the use Flumazenil in slow infusion in for BZD detoxification was still evaluated as experimental. The reason given by Soyka was that, although the technique had been described for more than 25 years, there were still too few centers in the world offering it (7, 17). This seems to have generated a vicious circle that has lasted for almost thirty years: a limited number of centers, albeit with numerous and well-documented cases and trials, but not sufficiently distributed to include the method in the guidelines, despite the truly impressive spread of prolonged BZD use, not infrequently with extra-therapeutic doses. A further demonstration of how much the use of high doses of BZD remains poorly studied and poorly defined is the difficulty of still finding an agreement on the definition of high dose (29).

FLU is commonly used in the treatment of BZD overdose, and it is usually considered a BZD antagonist. Nonetheless, results of studies in chronic BZD users who have discontinued BZDs suggest that multiple slow bolus infusions of FLU reduce the symptoms of withdrawal.

The role of FLU remains partially unclear. Its action may facilitate coupling of the GABA_*A*_ and BZD receptor complexes, potentially reversing the down regulation/uncoupling that occurs with long-term BZD misuse. This could explain its ability to attenuate withdrawal symptoms after chronic exposure to the drug, since it exercises weak agonistic action (13).

Apart from few previous anecdotal experiences, Gerra and colleagues were the first to propose a protocol and provide treatment in an outpatient HDUs hospital using FLU. Since then, apparently few centers around the world offer this BZD detoxification treatment (7). At present, FLU is indicated only in case of acute BDZ intoxication, recommending caution in chronic abusers to the risk of precipitating abstinence. The recent experience by FLU subcutaneous infusion increased the patient compliance and makes the treatment more “reassuring” for doctors, apparently removing barriers to treatment.

In spite of its potential benefits, some aspects of this therapeutic approach need to be clarified. With respect to the inclusion criteria, questions that could be posed include: the minimum diazepam-like BZD dosage threshold for recommending FLU treatment, and the minimum period of time of continuous use of BZD beyond where the FLU treatment can be recommended.

The times of administration and the end point of the treatment should also be better evaluated. With FLU being a partial agonist, it is likely that long-term administration will have a beneficial effect. Oral administration (not available on the market) has limited application due to the short half-life of the drug.

More studies are needed to determine the drug’s safety. Seizure risk is an important topic and remains one of the most relevant barriers among physicians. Underestimating these issues implies not allowing the use of the treatment on a larger scale. Related to this aspect, the opportunity to use adjunctive medications (e.g., anticonvulsants), in spite of encouraging data (11) needs to be explored further.

## Conclusion

Suggesting gradual tapering of the dose to HDUs patients is like suggesting that alcoholics gradually stop drinking: it simply doesn’t work, and the problem is aggravated by the fact that the tapering BZDs takes a lot of time. The crucial point is that these HDUs have been the subject of very little studies, and are virtually ignored in research and clinical practice, which tend to confine themselves to patients with co-addictions and personality disorders. But the situation is more complex. The capacity of BZDs to create tolerance, along with their low toxicity, can induce truly stupefying increments in dosage.

Flumazenil slow infusion works best with HDUs patients. Probably there have been too few scientific studies for it to be considered “good practice.” The aim of this paper is to promote this approach to become a treatment protocol. At this time perhaps the congress format, with a clearer emphasis on experience, would be the most appropriate way to promote this approach.

## Author Contributions

FL, RC, and AM were responsible for the study concept and design. LZ, MB, SC, and AM drafted the manuscript. All authors critically reviewed the content and approved the final version of the manuscript for publication.

## Conflict of Interest

AM was employed by Camden and Islington NHS Foundation Trust. The remaining authors declare that the research was conducted in the absence of any commercial or financial relationships that could be construed as a potential conflict of interest.

## Publisher’s Note

All claims expressed in this article are solely those of the authors and do not necessarily represent those of their affiliated organizations, or those of the publisher, the editors and the reviewers. Any product that may be evaluated in this article, or claim that may be made by its manufacturer, is not guaranteed or endorsed by the publisher.
