# Supplementary material for: Addiction of High Dose of Benzodiazepine: Verona Detox Approach With Flumazenil
Source: Front Psychiatry. 2022 Mar 31;13:857376. doi: 10.3389/fpsyt.2022.857376 (PMC9008883; doi:10.3389/fpsyt.2022.857376)
Supplement: Supplementary file 1 [file Data_Sheet_1.pdf]

Appendix 1

BZDs[1]	Half-life (hrs)[2] [active metabolite]	Approximately Equivalent Oral dosage (mg)[3]	Market aim[4]
Alprazolam (Xanax, Frontal, Valeans, Xanor, Tafil)	6-12	0,5	a
Bromazepam (Lexotan, Lexomil)	10-20	5-6	a
Chlordiazepoxide (Librium)	5-30 [36-200]	25	a
Clobazam (Frisium)[5]	12-60	20	a,e
Clonazepam (Rivotril, Klonopin)[5]	18-50	0,5	a,e
Clorazepate (Tranxene)	[36-200]	15	a
Diazepam (Valium, Tranquirit, Ansiolin)	20-100 [36-200]	10	a
Estazolam (Esilgan, ProSom, Nuctalon)	10-24	1-2	h
Flunitrazepam (Roi hypnol)	18-26 [36-200]	1	h
Flurazepam (Felison, Dalmadorm, Dalmane)	[40-250]	15-30	h
Halazepam (Paxipam)	[30-100]	20	a
Ketazolam (Anseren, Anxon)	30-100 [36-200]	15-30	a
Loprazolam (Dormonoct)	6-12	1-2	h
Lorazepam (Tavor, Ativan, Temestra)	10-20	1	a
Lormetazepam (Minias, Noctamid)	10-12	1-2	h
Medazepam (Nobrium)	36-200	10	a
Nitrazepam (Mogadon)	15-38	10	h
Nordazepam (Madar, Nordaz, Calmday)	36-200	10	a
Oxazepam (Serpax, Serax, Serenid, Seresta)	4-15	20	a
Prazepam (Prazene, Centrax, Lysanxia)	[36-200]	10-20	a
Quazepam (Doral, Quazium)	25-100	20	h
Temazepam			

(Normison, Restoril, Euhypnos)	8-22	20	h
Triazolam (Halcion)	2	0,5	h
Non-BZDs with similar effects[1, 6]			
Zaleplon (Sonata)	2	20	h
Zolpidem (Stilnox, Sonirem, Ambien, Stilnoct)	2	20	h
Zopiclone (Imovane, Zimovane)	5-6	15	h
Eszopiclone (Lunesta)	6 (9 in elderly)	3	h

APPENDIX 2: BENZODIAZEPINE WITHDRAWAL SCALE (CIWA-B)

DRUG & ALCOHOL SERVICES

BENZODIAZEPINE WITHDRAWAL SCALE (CIWA-B) 1

CLIENT SHEET

Surname:
Other names:
DOB: Sex: M F UR
Number:

For each of the following items, insert the number that best describes how you feel.	0<br>Not at all	1	2	3	4<br>Very much so

DATE														
TIME														
1 Do you feel irritable?														
2 Do you feel fatigued?														
3 Do you feel tense?														
4 Do you have difficulties concentrating?														
5 Do you have any loss of appetite?														
6 Have you any numbness or burning in your face, hands or feet?														
7 Do you feel your heart racing? (palpitations)														
8 Does your head feel full or achy?														
9 Do you feel muscle aches or stiffness?														
10 Do you feel anxious, nervous or jittery?														
11 Do you feel upset?														
12 How restful was your sleep last night? (0 = very much so; 4 = not at all)														
13 Do you feel weak?														
14 Do you think you had enough sleep last night? (0 = very much so; 4 = not at all)														
15 Do you have any visual disturbances? (sensitivity to light, blurred vision)														
16 Are you fearful?														
17 Have you been worrying about possible misfortunes lately?														
SUB-TOTAL														

BENZODIAZEPINE WITHDRAWAL SCALE (CIWA-B) 2

CLINICIAN SHEET

Surname:
Other names:
DOB: Sex: M F UR
Number:

Clinician observations

18. Observe behaviour for sweating, restlessness and agitation	
0	None, normal activity
1	
2	Restless
3	
4	Paces back and forth, unable to sit still

19. Observe tremor	
0	No tremor
1	Not visible, can be felt in fingers
2	Visible but mild
3	Moderate with arms extended
4	Severe, with arms not extended

20. Observe feel palms	
0	No sweating visible
1	Barely perceptible sweating, palms moist
2	Palms and forehead moist, reports armpit sweating
3	Beads of sweat on forehead
4	Severe drenching sweats

TIME/DATE														
AGITATION														
TREMOR														
SWEATING														
CLIENT'S SCORE (from pg 1)														
TOTAL SCORE														
BLOOD PRESSURE														
PULSE														
TEMPERATURE per axilla														
RESPIRATIONS														
ALERT, ORIENTATED, OBEYS COMMANDS? *If NO, complete GCS* and review.*	Y / N	Y / N	Y / N	Y / N	Y / N	Y / N	Y / N	Y / N	Y / N	Y / N	Y / N	Y / N	Y / N	Y / N

PUPIL SIZE/REACTION (in mm)	L																												
	R																												

TOTAL SCORE FOR ITEMS 1 - 20

1-20 = mild withdrawal 21-40 = moderate withdrawal 41-60 = severe withdrawal 61 - 80 = very severe withdrawal

**Glasgow Coma Scale*

Brief Substance Craving Scale

Agency Name: ______________________ **Site Name:** ______________________

ID #: ____________________ **Date:** __ __ / __ __ / __ __ __ __

STAFF USE ONLY

A. Identify the primary substance dependence for which the participant is being treated at this clinic.

- Downers or Sedatives (Barbiturates, etc.) ❑1
- Benzos (Valium, Xanax, etc.) ❑2
- Hallucinogens (including ecstasy) ❑3
- Alcohol ❑4
- Heroin or other Opiates (Morphine, etc.) ❑5
- Marijuana ❑6
- Stimulants (cocaine, amphetamine) ❑7
- Other (specify): ______________________ ❑8

Please answer the following questions with regard to your craving for the primary drug.

1. The INTENSITY of my craving, that is, how much I desired this drug in the past 24 hours was:

- None at all ❑0
- Slight ❑1
- Moderate ❑2
- Considerable ❑3
- Extreme ❑4

2. The FREQUENCY of my craving, that is, how often I desired this drug in the past 24 hours was:

- Never ❑0
- Almost never ❑1
- Several times ❑2
- Regularly ❑3
- Almost constantly ❑4

3. The LENGTH of time I spent in craving this drug during the past 24 hours was:

- None at all ❑0
- Very short ❑1
- Short ❑2
- Somewhat long ❑3
- Very long ❑4

4. Write in the NUMBER of times you think you had craving for this drug during the past 24 hours. ___ ___

ID #: ________________________ Date: ___ ___ / ___ ___ / ___ ___ ___ ___

B. A second craved substance during the past 24 hours was:
Choose only ONE from the following. If NONE, please do not answer Questions 5-8.

- **None (STOP)** ☐0
- Downers or Sedatives (Barbiturates, etc.) ☐1
- Benzos (Valium, Xanax, etc.) ☐2
- Hallucinogens (including ecstasy) ☐3
- Alcohol ☐4
- Heroin or other Opiates (Morphine, etc.) ☐5
- Marijuana ☐6
- Stimulants (cocaine, amphetamine) ☐7
- Other (specify): ______________________________ ☐8

Please answer the following questions with regard to a second craved drug.

5. The INTENSITY of my craving, that is, how much I desired this drug in the past 24 hours was:

- None at all ☐0
- Slight ☐1
- Moderate ☐2
- Considerable ☐3
- Extreme ☐4

6. The FREQUENCY of my craving, that is, how often I desired this drug in the past 24 hours was:

- Never ☐0
- Almost never ☐1
- Several times ☐2
- Regularly ☐3
- Almost constantly ☐4

7. The LENGTH of time I spent in craving this drug during the past 24 hours was:

- None at all ☐0
- Very short ☐1
- Short ☐2
- Somewhat long ☐3
- Very long ☐4

8. Write in the NUMBER of times you think you had craving for this drug during the past 24 hours. ___ ___

Reference: Somoza, E., Dyrenforth, S., Goldsmith, J., Mezinskis, J., & Cohen, M., 1995. In search of a universal drug craving scale. Paper presented at the Annual Meeting of the American Psychiatric Association, Miami Florida.

APPENDIX 4: FIRST AID FOR SEIZURES

First aid for epileptic seizures

Remember

ACTION

for tonic-clonic seizures:

A — **Assess**
Assess the situation – are they in danger of injuring themselves? Remove any nearby objects that could cause injury

C — **Cushion**
Cushion their head (with a jumper, for example) to protect them from head injury

T — **Time**
Check the time – if the jerking lasts longer than five minutes you should call an ambulance

I — **Identity**
Look for a medical bracelet or ID card – it may give you information about the person's seizures and what to do

O — **Over**
Once the jerking has stopped, put them on their side. Stay with them and reassure them as they come round

N — **Never**
Never restrain the person, put something in their mouth or try to give them food or drink

Tonic-clonic seizures

(used to be called 'grand mal')
The person goes stiff, loses consciousness, falls to the floor and begins to jerk or convulse. They may look a little blue around their mouth from irregular breathing. Tonic-clonic seizures can last a few minutes. A tonic-clonic seizure is the seizure you are most likely to come across. There are many others. Visit epilepsy.org.uk to find out more.

Call an ambulance if:

- You know it is a person's first seizure **or**
- The seizure lasts for more than five minutes **or**
- One seizure appears to follow another without the person gaining consciousness in between **or**
- The person is injured **or**
- You believe the person needs urgent medical attention

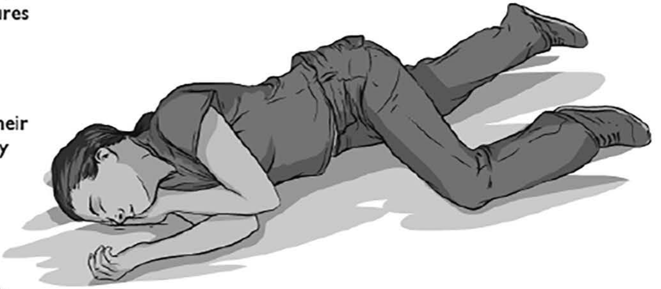

B148B.02
Date: July 2015
Reprinted: May 2016
Due for review: July 2018

Epilepsy Action
New Anstey House, Gate Way Drive, Yeadon, Leeds LS19 7XY
tel. 0113 210 8800 email epilepsy@epilepsy.org.uk epilepsy.org.uk
Epilepsy Action Helpline: freephone 0808 800 5050 text 07797 805 390
email helpline@epilepsy.org.uk twitter @epilepsyadvice

Epilepsy Action is a working name of British Epilepsy Association. A company limited by guarantee (registered in England No. 797997)
Registered charity in England (No. 234343)

Health & care information you can trust
The Information Standard Certified Member

© Copyright Epilepsy Action 2016
